# Effect of Nutrition Interventions on the Dietary Diversity Status Among Pregnant Women in Ethiopia: Systematic Review and Meta‐Analysis

**DOI:** 10.1002/fsn3.71231

**Published:** 2025-11-28

**Authors:** Mahider Awoke Belay, Mulat Belay Simegn, Samuel Dagne Chanie, Werkneh Melkie Tilahun, Yonatan Menber, Yosef Wasihun, Zenebe Abebe Gebreegziabher, Zewudu Andualem, Ayenew Takele Alemu, Azeb Geddif, Genet Gedamu Kassie, Kalkidan Worku Mitiku

**Affiliations:** ^1^ Department of Public Health, College of Medicine and Health Sciences Inibara University Injibara Ethiopia; ^2^ Department of Public Health, College of Medicine and Health Sciences Debre Markos University Debre Markos Ethiopia; ^3^ Department of Nutrition and Dietetics, School of Public Health, College of Medicine and Health Sciences Bahir Dar University Bahir Dar Ethiopia; ^4^ Department of Health Promotion and Behavioral Sciences, School of Public Health, College of Medicine and Health Sciences Bahir Dar University Bahir Dar Ethiopia; ^5^ Department of Epidemiology and Biostatistics, School of Public Health, Asrat Woldeyes Health Science Campus Debre Berhan University Debre Berhan Ethiopia; ^6^ Department of Environmental and Occupational Health and Safety, Institute of Public Health, College of Medicine and Health Sciences University of Gondar Gondar Ethiopia; ^7^ Department of Environmental Health, School of Public Health, College of Medicine and Health Sciences Bahir Dar University Bahir Dar Ethiopia; ^8^ Department of Pediatrics and Child Health Nursing, School of Nursing and Midwifery, College of Medicine and Health Sciences Bahir Dar University Bahir Dar Ethiopia

**Keywords:** dietary diversity, Ethiopia, meta‐analysis, nutrition intervention, pregnant women, systematic review

## Abstract

The government of Ethiopia recommends nutrition counseling throughout pregnancy for better consumption of diversified diets, because improving maternal nutrition is vital for the prevention of maternal and child mortality and morbidity. As a result, the purpose of this study aimed to summarize the national evidence on the effect of nutrition intervention on dietary diversity status among pregnant women in Ethiopia using a systematic review and meta‐analysis. The advanced search of electronic databases and search engines was used to conduct a systematic review and meta‐analysis of the effect of nutrition intervention on the dietary diversity status among pregnant women in Ethiopia. Data extraction and quality appraisal were done through the standardized JBI tool. STATA V.17 was used for analysis. The meta‐analysis was carried out using the random effects model. *I*‐squared and *Q*‐tests were used to detect the heterogeneity of the findings. A total of 3891 participants were included in this systematic review and meta‐analysis study from 9 and 8 articles, respectively. The pooled difference in difference percentage and mean effects of nutrition intervention on the dietary diversity status among pregnant women were 35.63% (95% CI; 6.87%, 64.39%) and 0.78 (95% CI; 0.38, 1.18), respectively. The heterogeneity tests were very high (*I* ^2^ = 99.65%, *p* = 0.00, and 99.96%, *p* = 0.00) for pooled percentage and mean, respectively. The pooled difference in difference percentage effect of nutrition intervention on the dietary diversity status among the pregnant women from the other regions was higher (57.69% (95% CI: 23.88%, 91.50%)) than the Oromia region (13.63% [95% CI: 3.54%, 23.73%]). The pooled percentage of the effect of nutrition intervention on the dietary diversity status among pregnant women who had received equal to or greater than 5 months intervention was high (44.70% [95% CI: 23.46%, 106.87%]) as compared to the corresponding (29.61% [95% CI: 8.35%, 50.88%]). Nutritional interventions for pregnant women had a positive effect on dietary diversity status. Therefore, efforts should be made to promote nutrition intervention utilizing the HBM and TPB, targeting couples in constructing nutrition education interventions.

## Graphical abstract

A total of 3891 participants from 9 and 8 articles were included in this SR & MA, respectively. The pooled DID percentage and mean effects of nutrition intervention on DDS among pregnant women were 35.63% (95% CI; 6.87%, 64.39%) and 0.78 (95% CI; 0.38, 1.18), respectively. Nutritional interventions for pregnant women have a positive effect on dietary diversity status. Therefore, efforts should be made to promote nutrition intervention utilizing the HBM and TPB, targeting couples in constructing nutrition education interventions.

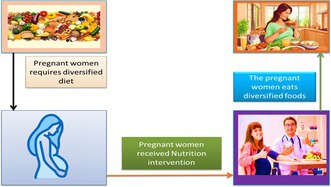

**Abbreviations**
ANC: anti‐natal care
DDS: dietary diversity score
DID: difference in difference
HBM: health belief model
HINARI: health internetwork access to research initiative
JBI: Joanna Briggs Institute
PRISMA: preferred reporting items for systematic reviews and meta‐analysis
TPB: theory of planned behavior

## 1 Introduction

Dietary diversity has also been used to indicate nutrient adequacy and the quality of dietary consumption, specifically the likelihood of micronutrient adequacy in a diet. It is a qualitative measure of food consumption that reflects a person's availability to a wide variety of foods (Kennedy et al. 2011). Dietary diversity score (DDS) was computed by adding the number of food groups eaten by each respondent over the previous 24 h (Kennedy et al. 2011). Women of reproductive age (WRA) living in low‐ and middle‐income countries (LMIC) are especially vulnerable to malnutrition, notably in key micronutrients (Torheim et al. 2010). Adequate intakes of important micronutrients, a balanced and proper supply of macronutrients, and energy are required to meet the physiological demands of the growing fetus, improve her nutritional profile, and lower the risk of maternal and child mortality and morbidity (Wu et al. 2012). A pregnant woman with a limited diet is more likely to be low in essential nutrients, depriving the fetus of the nutrition it requires for proper growth (Sebastiani et al. 2019).

Many women in sub‐Saharan Africa, especially in Ethiopia, continue to have insufficient micronutrient intake, leading to various types of malnutrition and consequences. Low maternal dietary diversity consumption is responsible for 7% of the worldwide disease burden, one‐fifth of maternal deaths, and poor maternal outcomes (Harika et al. 2017; Mudogo 2017; Haque et al. 2023). The pooled prevalence of inadequate dietary diversity scores among pregnant women in Ethiopia was 53% (Hidru et al. 2020). Studies done in Ethiopia, in Shashemane 75% (Desta et al. 2019), Dire Dawa 57% (Shenka et al. 2018), Ambo 73.6% (Gebremichael and Belachew Lema 2023a), and Addis Ababa 51.6% (Kebede et al. 2022), revealed that pregnant women had inadequate dietary diversity intake.

Dietary diversity in pregnant women is not only important for lowering maternal mortality but also for promoting fetal growth and avoiding perinatal outcome complications. Inadequate dietary diversity intake during pregnancy leads to increased risks such as intrauterine growth restriction (IUGR), preterm birth, low birth weight, abortion, prenatal and neonatal mortality, and morbidity (Wu et al. 2012; Marshall et al. 2022; Cano‐Ibáñez et al. 2020; Wondemagegn et al. 2022).

Different research revealed that food insecurity, family size, residence, educational status, livestock ownership, poor socioeconomic status, women who received emotional support from their husbands and who participated in shopping, empowered women, produced legumes and nuts, knowledge about dietary diversity, and a lack of counseling about dietary diversity were the significant factors for inadequate dietary diversity status among pregnant women (Hidru et al. 2020; Desta et al. 2019; Kebede et al. 2022; Hossain et al. 2024).

The World Health Organization provides recommendations for maternal and child nutrition throughout their lifetimes. It emphasizes direct nutrition interventions provided by health professionals who provide nutrition education and/or counseling for better consumption of diversified diets, which have a significant impact on nutrition (World Health Organization 2013). The government of Ethiopia recommends nutrition counseling throughout pregnancy (Federal Ministry of Health 2016) because improving maternal nutrition is vital for the prevention of maternal and child mortality and morbidity (Omer et al. 2020; Zelalem et al. 2017). In Ethiopia, health extension workers and health specialists provide nutrition counseling during anti‐natal care (ANC) follow‐up and at the community level, with a focus on the necessity for pregnant women to eat one extra meal (Demilew et al. 2020).

Some studies were undertaken in Ethiopia to investigate the effects of nutrition interventions on pregnant women's dietary diversity (Omer et al. 2020; Demilew et al. 2020; Sisay and Tesfaye 2023; Beressa et al. 2024; Tsegaye et al. 2022, 2023; Kuma et al. 2023; Gebremichael and Belachew Lema 2023b; Tesfaye et al. 2025). As a result, this study aimed to summarize the national evidence on the effect of nutrition intervention on dietary diversity status among pregnant women in Ethiopia using a systematic review and meta‐analysis.

## 2 Methods and Materials

### 2.1 Data Sources and Searching Strategies

**FIGURE 1:**
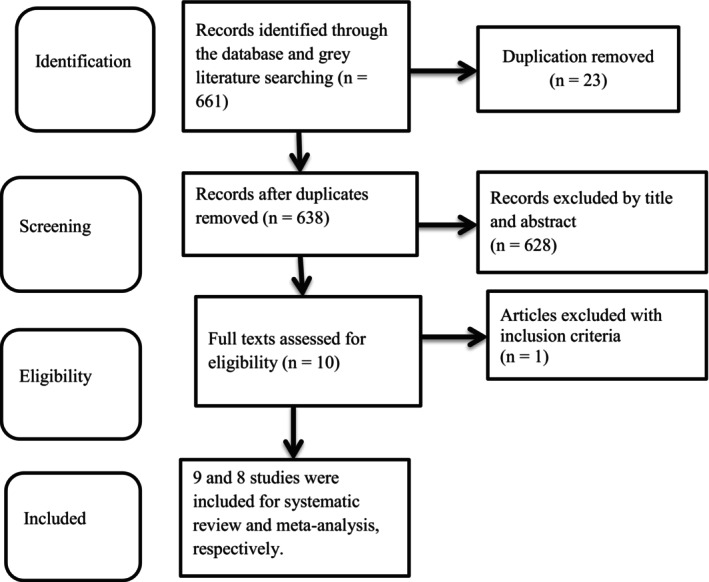
Flow diagrams of the studies included in the review of the effects of nutrition intervention on the DDS among pregnant women in Ethiopia.

The protocol for the systematic review and meta‐analysis was registered under the registration ID CRD42024539357 with PROSPERO (International Prospective Register of Systematic Review and Meta‐analysis). The whole systematic review and meta‐analysis was written using the Preferred Reporting Items for Systematic Reviews and Meta‐analysis (PRISMA) principles of statement (Figure 1) (Page et al. 2021). The strategy of searching was aimed at identifying both published and unpublished studies. Searching of the literature was carried out utilizing the Cochrane Library, PubMed, and Health Inter Network Access to Research Initiative bibliographic databases (HINARI). Gray literature was obtained from Google and Google Scholar search engines. Searching was conducted from April 10 to 16, 2025. Articles that couldn't be found in databases were examined by contacting experts and communication addresses.

To obtain the required studies, search terms were developed. The following search keywords were entered: “nutrition intervention,” “nutrition education,” “nutrition counseling,” “behavioral change communication,” “dietary diversity status*,” “dietary diversity score*,” “dietary consumption,” “home gardening,” “pregnant women*,” “pregnant mother*,” and “Ethiopia.” Boolean operators (“AND” and “OR”) and truncation (“*”) were applied in advanced database searching.

### 2.2 Study Selection (Eligibility Criteria) and Screening

This review was restricted to interventional studies only.

**Inclusion Criteria:** Interventional studies conducted in Ethiopia about the effects of nutrition intervention (nutrition and health behavior change communication, nutrition education, and/or counseling) on the dietary diversity status of pregnant women; published or unpublished articles were included.

**Exclusion Criteria:** Studies with no full texts and languages without English were excluded.

**Outcome:** The effects of intervention (difference in change in intervention and control group) were reported in difference in differences (DID).

Retrieved studies from various databases were imported into EndNote version 20.2, and duplicated articles were removed upon detection. The remaining articles were screened through titles and abstracts by the two independent reviewers (MAB and MBS), and the full text was also evaluated by another two independent reviewers (SDC and WMT) against the inclusion criteria.

### 2.3 Data Extraction Process

Data were extracted by a standardized data extraction format, derived from the JBI on a Microsoft Excel spreadsheet, through the two independent reviewers (YM and YW) (Munn et al. 2014; Bui et al. 2016). The extracted data included specific details of authors, year of publication, sample size, region, participants, significant factors, length of intervention, and overall effects of intervention. If there was any dissimilarity between the two reviewers while screening, it was resolved through discussion or by an involved 3rd reviewer.

### 2.4 Quality Appraisal and Risk of Bias in the Studies

The two independent reviewers (ZAG and ZA) critically appraised the eligible studies before inclusion. The quality of every article was evaluated using the standardized Joanna Briggs Institute (JBI) critical evaluation method for experimental studies (Barker, Habibi, et al. 2024; Barker, Hasanoff, et al. 2024). The quality of the results was also evaluated by two other independent reviewers (ATA and AG) to check their consistencies. The results were totaled and converted to a percentage. For the meta‐analysis and systematic review of prevalence, only studies with a score of 70% and above were taken into account (Barker, Habibi, et al. 2024; Barker, Hasanoff, et al. 2024). Any discrepancies in the evaluators' scores were examined by performing a comprehensive revision, and conflicts were settled by dialoge. Funnel plots and Egger's tests were used to assess the publication bias.

### 2.5 Synthesis of Results

The retrieved data were imported to STATA V.17 for additional analysis. The effects of nutrition intervention were pooled using the random effect model with the restricted maximum likelihood (REML) method. Heterogeneity was evaluated using the *I*‐square test and *Q*‐test. Values of *I*‐square above 75% and a *p* < 0.05 were considered indicative of significant heterogeneity. From the interpretation, there was high, medium, and low heterogeneity present at *I* ^2^ values of 75%, 50%, and 25%, respectively (Thorlund et al. 2012). Analyzes of subgroups and sensitivity were used to investigate potential causes of heterogeneity. Tables, texts, and forest plots were used to display the results.

## 3 Results

### 3.1 Study Search and Selection

During the database search, a total of 661 articles were retrieved from PubMed (410), the Cochrane Library (45), HINARI (10), Google Scholar (150), and Google (46) (Figure 1) from April 10 to 16, 2025. From this study, 23 duplicate results were removed, and 628 articles were excluded after screening by the titles and abstracts of the study. Finally, we assessed the full texts of 10 records for eligibility, and one study was excluded by the inclusion criteria (full text not available) (Negash et al. 2023). Nine and 8 articles were considered for systematic review and meta‐analysis, respectively, which were conducted in Amhara (Demilew et al. 2020), Oromia (Beressa et al. 2024; Tsegaye et al. 2022, 2023; Kuma et al. 2023; Gebremichael and Belachew Lema 2023b; Tesfaye et al. 2025), Addis Ababa (Omer et al. 2020) and Southern Nation and Nationalities of People Representatives (SNNPR) (Sisay and Tesfaye 2023). Four studies were reported in percentage (Omer et al. 2020; Demilew et al. 2020; Beressa et al. 2024; Gebremichael and Belachew Lema 2023b) and mean (Sisay and Tesfaye 2023; Tsegaye et al. 2022, 2023; Tesfaye et al. 2025); the rest reported in beta coefficient (Kuma et al. 2023). Therefore there were two pooled effects in this study (percentage and mean). Three studies contained the two eligible outcome variables, which were conducted in the Jimma and Illu Aba Bor zones in the Oromia region, which had two intervention groups (couples and women alone and peers and women alone, respectively) and a control group (Tsegaye et al. 2022, 2023; Kuma et al. 2023). Some of the studies used nutrition education (Beressa et al. 2024; Tsegaye et al. 2022; Kuma et al. 2023; Sisay and Tesfaye 2023), two studies used nutrition and health behavioral change communication (NHBCC) (Gebremichael and Belachew Lema 2023b; Tesfaye et al. 2025), and others used nutrition counseling (Demilew et al. 2020; Sisay and Tesfaye 2023) for intervention (Table 1). A total of 3891 participants were involved in this study. Three studies employed ten food groups (Kuma et al. 2023; Tesfaye et al. 2025; Sisay and Tesfaye 2023), two studies did not clearly specify (Omer et al. 2020; Gebremichael and Belachew Lema 2023b), and four studies used nine food groups for DDS assessment (Demilew et al. 2020; Beressa et al. 2024; Tsegaye et al. 2022, 2023).

**TABLE 1:** The characteristics of the included studies in the systematic review and meta‐analysis of the effects of nutrition intervention on the DDS of pregnant women in Ethiopia.

The first author's	Region	Publication year	Study design	Sample size	Duration of intervention	Intervention groups	Control groups	Overall effects of the intervention
Demilew YM et al.	Amhara	2020	A two‐arm parallel cluster randomized controlled community trial	645	4 months	Attended 4 nutrition counseling sessions.	Attended routine nutrition education only	Pregnant women in the intervention group had adequate DDS, FVS, high animal source food consumption, and adequate food frequency compared to the control group. The DID effect of nutrition intervention on DDS was 40.5%.
Kuma MN et al.	Oromia	2023	A three‐parallel‐arms, community‐based, cluster randomized controlled trial	348	9 months	Three nutrition education sessions and four varieties of vegetable seeds were provided for women in the intervention arms (husband and peers).	Attended routine nutrition education only (usual services).	In the intervention arms (husband and peer), pregnant women born to neonates with heavier weight, high bands of hemoglobin, and high minimum dietary diversity scores improved nutritional knowledge and dietary practice more than the control groups. The effect of nutrition intervention on the DDS was β = 0.87 and 0.22 for the husband and peer, respectively.
Tsegaye et al.	Oromia	2022	Pretest‐posttest quasi‐experimental study	403	3 months	The couple and pregnant women alone received 3 nutrition education sessions.	No received nutrition education	The intervention arms (couple (husband and wife) and Women) had positive effects on the nutritional status and dietary diversity status of pregnant women. The mean DID effects of intervention on DDS were 0.85 ± 0.19 and 0.44 ± 0.21 for the couple and women‐alone groups, respectively.
Beressa et al.	Oromia	2024	Cluster randomized controlled trial	447	6 months	Received six nutrition education counseling sessions and a structured work schedule.	Received only routine nutrition education at a health facility	The intervention group had a higher DDS than the control. The wealth index, educational status, and received intervention were factors that affected the DDS. The effect of nutrition intervention on DDS was 8.5%.
Sisay G. and Tesfaye A.	SNNPR	2021	Cluster randomized controlled trial	235	6 months	Received 4 sessions of nutrition education and counseling.	Received routine nutrition counseling (usual services)	The effect of intervention improved maternal nutritional status and child birth weight. The overall mean difference effect of nutrition intervention on DDS was 0.27 ± 0.1.
Gebremichael MA. and Belachew LT.	Oromia	2023	A two‐arm parallel cluster randomized controlled community trial	744	3 months	Received six NHBCC sessions.	Did not receive the NHBCC intervention	The overall effect of nutrition and behavior change communication through community‐level actors improved the nutritional status of pregnant women. The overall effect of nutrition intervention on DDS was 18.8%.
Omer AM. et al.	Addis Ababa	2020	A cluster randomized controlled trial	240	5 months	Pregnant women received nutrition education from trained health care providers.	Not attended the nutrition education and counseling packages.	The effect of the comprehensive in‐service nutrition education and counseling package on health care providers improving the knowledge of health‐care workers and their skills in pregnancy nutrition also improved the DDS, meal frequency, and weight gain during pregnancy. The DID effect of intervention on DDS among pregnant women Was 75%.
Tesfaye A et al.	Oromia	2025	A two‐arm parallel cluster randomized controlled trial	426	9 months	The pregnant adolescent and husband received 4 NBCC sessions.	Received standard nutrition counseling (usual services)	The nutrition intervention improved DDS, dietary practice, meal frequency, and nutritional knowledge of the pregnant adolescent. The DID mean effect intervention on the DDS was 1.69 ± 0.3.
Tsegaye D. et al.	Oromia	2022	Pretest‐posttest quasi‐experimental	403	3 months	The couple and pregnant women alone received 3 nutrition education sessions.	Received standard nutrition counseling (usual services)	The DID mean effects of intervention on the DDS were 0.8 ± 0.09 and 0.62 ± 0.46 for couples and women‐alone, respectively.

### 3.2 The Effects of Nutrition Intervention on the Dietary Diversity Status Among Pregnant Women

**FIGURE 2:**
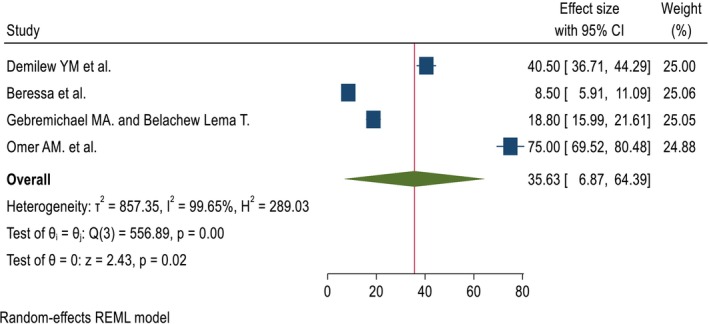
The pooled DID percentage effects of nutrition intervention on the DDS status among pregnant women in Ethiopia.

**FIGURE 3:**
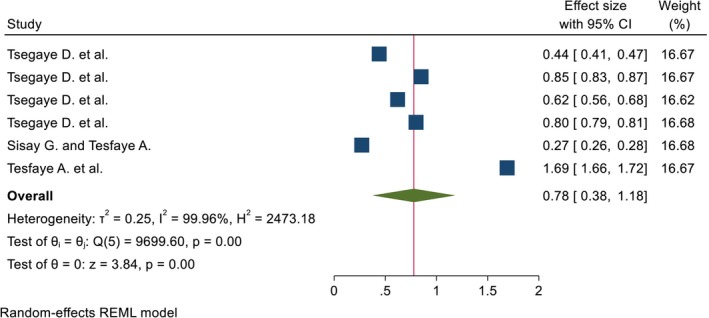
The pooled DID mean effects of nutrition intervention on the DDS status among pregnant women in Ethiopia.

**FIGURE 4:**
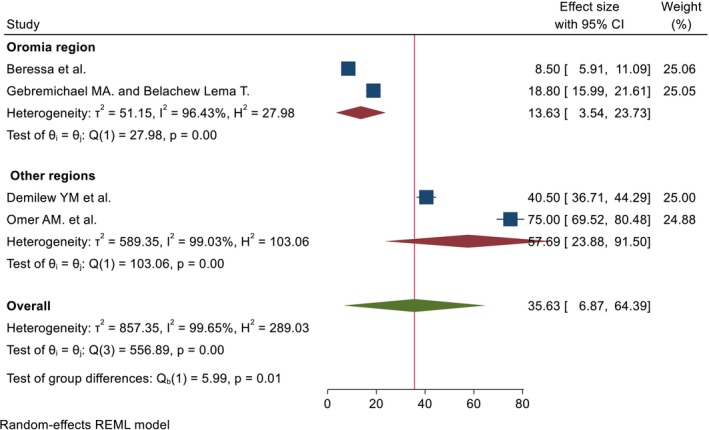
Subgroup analysis by the region of the pooled DID percentage effects of nutrition intervention on the DDS among pregnant women in Ethiopia.

**FIGURE 5:**
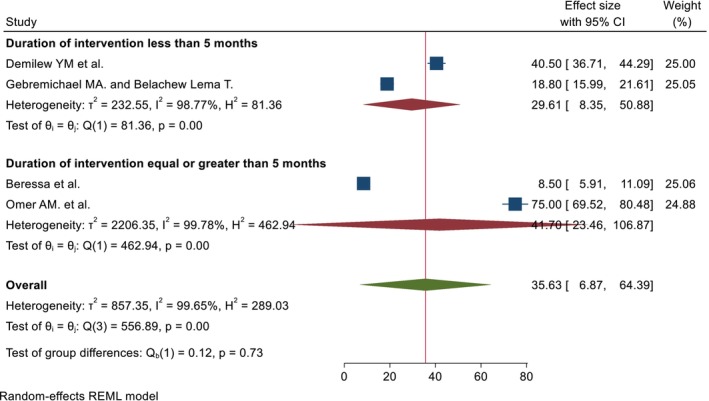
Subgroup analysis by the duration of intervention of the pooled DID percentage effects of nutrition intervention on the DDS among pregnant women in Ethiopia.

**FIGURE 6:**
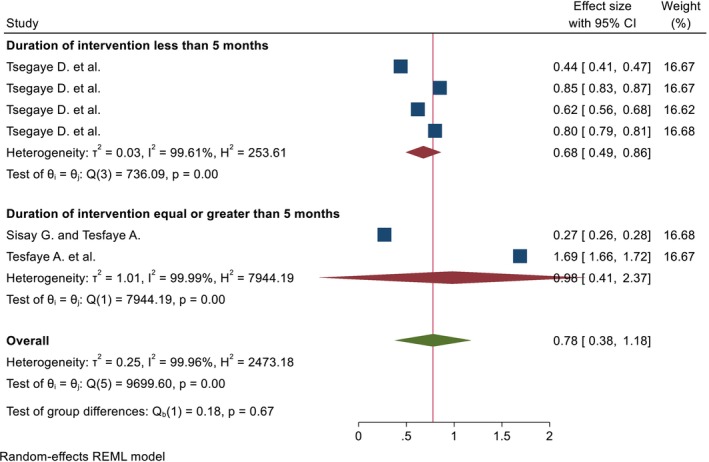
Subgroup analyzes by the duration of intervention of the pooled DID mean effects of nutrition intervention on the DDS among pregnant women in Ethiopia.

A total of 4 articles were used to estimate the pooled percentage, and 4 papers with 6 outcomes were used to estimate the pooled mean. The pooled percentage and mean DID effect of nutrition intervention on the DDS among pregnant women was 35.63% (95% CI; 6.87%, 64.39%) and 0.78 (95% CI; 0.38, 1.18), respectively. The heterogeneity tests were very high (*I* ^2^ = 99.65%, *p* = 0.00, and 99.96%, *p* = 0.00) for pooled percentage and mean DID, respectively (Figures 2 and 3). Due to the presence of high heterogeneity, subgroup analysis was done based on region and duration of intervention. The pooled DID percentage effect of nutrition intervention on the DDS among the pregnant women from the Other (Amhara and Addis Ababa) regions was higher (57.69% (95% CI: 23.88%, 91.50%)) than the Oromia region (13.63% (95% CI: 3.54%, 23.73%)). The heterogeneity test was very high (*I* ^2^ = 99.03%, *p* = 0.00, and *I* ^2^ = 96.43%, *p* = 0.00) from the Other and Oromia regions, respectively (Figure 4). Regarding the duration of intervention, the pooled DID percentage effect of nutrition intervention on the DDS among pregnant women who had received equal to or greater than 5 months was high (44.70% (95% CI: 23.46%, 106.87%)) as compared to the duration of intervention less than 5 months (29.61% (95% CI: 8.35%, 50.88%)). The heterogeneity test was also very high for both categories (*I* ^2^ = 97.78%, *p* = 0.00, and *I* ^2^ = 98.77%, *p* = 0.00) of equal to or greater than 5 and less than 5 months, respectively (Figure 5). Pregnant women who had received an intervention for 5 months and above have a higher pooled DID mean effect (0.98 (95% CI; 0.41, 2.37)) than the corresponding (0.68 (95% CI; 0.49, 0.89)) (Figure 6).

### 3.3 Quality Appraisal, Publication Bias, and Sensitivity Tests

**FIGURE 7:**
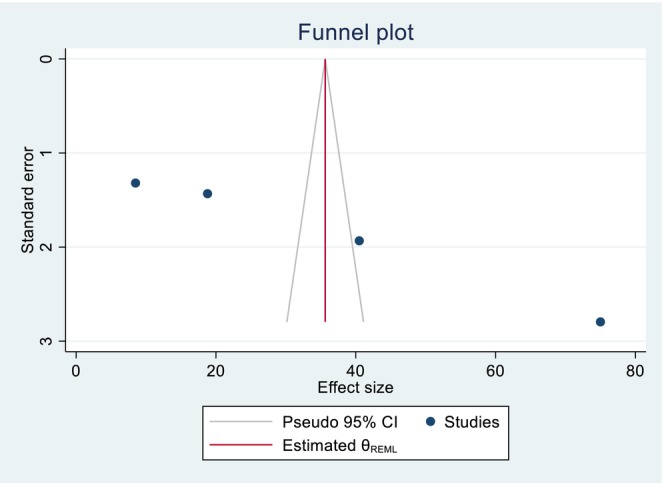
Symmetric presentation of the funnel plot graph for the pooled DID percentage effects of nutrition intervention on the DDS among pregnant women in Ethiopia.

**FIGURE 8:**
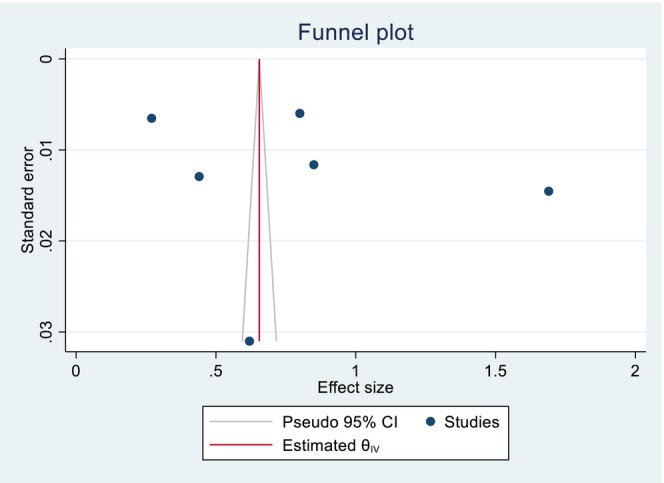
Publication biases of the funnel plot graph for the pooled DID mean effects of nutrition intervention on the DDS among pregnant women in Ethiopia.

**FIGURE 9:**
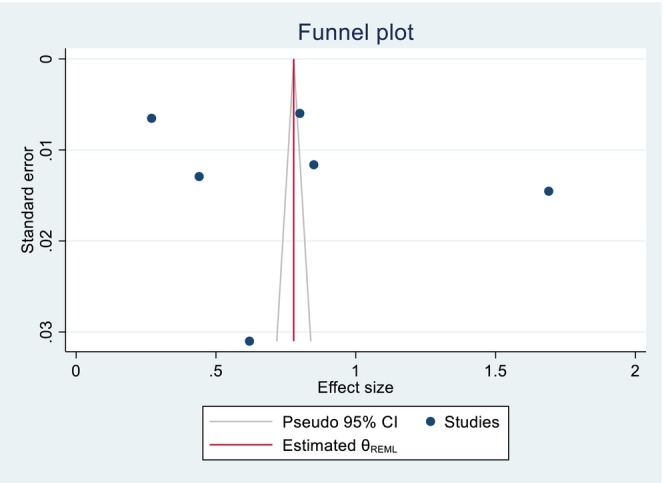
Symmetric presentation of the data, after the fill and trim techniques of analysis of publication bias for the pooled DID mean effects of nutrition intervention on the DDS among pregnant women in Ethiopia.

**FIGURE 10:**
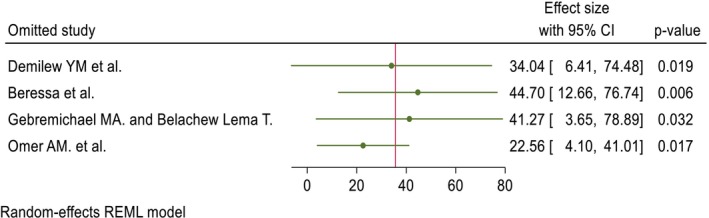
The sensitivity test of the pooled DID percentage effects of nutrition intervention on the DDS of pregnant women in Ethiopia.

**FIGURE 11:**
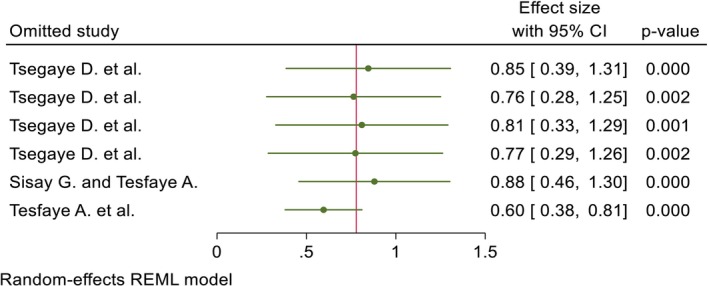
The sensitivity tests of the pooled DID mean effects of nutrition intervention on the DDS of pregnant women in Ethiopia.

The included studies of quality scores were above 80%, which was assessed by the two independent reviewers (GGK and KWM). The publication bias was identified through a funnel plot and Egger's test. Regarding the pooled DID percentage result, there was symmetric presentation of the funnel plot, and the Egger's test shows significance (Prob > |z| = 0.0000), which indicates that there was no publication bias (Figure 7; Table 2). Concerning the pooled DID mean report, an asymmetric distribution and non‐significant result were reported by the funnel plot and Egger's test (Prob > |*z*| = 0.8857), respectively, which implies the presence of publication bias (Figure 8; Table 3). The fill and trim techniques of analysis were employed to reduce the publication bias, with the imputation of zero (0) studies. The funnel plot looks symmetric after imputation (Figure 9). The sensitivity analysis of the included studies indicated that there were no findings that affected the pooled DID percentage and mean and the *p*‐value also remained significant (Figures 10 and 11).

**TABLE 2:** Regression‐based Egger test for small study effects, objectively showing the presence of publication bias for the pooled DID percentage effects of nutrition intervention on the DDS among pregnant women in Ethiopia.

Random effect REML model	
H0: beta	0; no small study effects
beta1	43.95
SE of beta1	3.153
*Z*	13.94
Prob >|*z*|	0.0000

**TABLE 3:** Regression‐based Egger test for small study effects, objectively showing the presence of publication bias for the pooled DID mean effects of nutrition intervention on the DDS among pregnant women in Ethiopia.

Random effect REML model	
H0: beta	0; no small study effects
beta1	3.91
SE of beta1	27.180
*Z*	0.14
Prob >|*z*|	0.8857

## 4 Discussion

The pooled percentage and mean DID effects of nutrition intervention on the DDS among pregnant women were 35.63% (95% CI; 6.87, 64.39) and 0.78 (95% CI; 0.38, 1.18), respectively. The heterogeneity tests were very high (*I* ^2^ = 99.65%, *p* = 0.00, and 99.96%, *p* = 0.00) for pooled percentage and mean DID, respectively. The occurrence of great heterogeneity may be attributed to the small number of studies, variance in the magnitude of the outcome variable, study season, and intervention status.

The pooled DID percentage effects of nutrition intervention on the DDS among the pregnant women in the Amhara and Addis Ababa regions was higher (57.69% (95% CI: 23.88, 91.50)) than in the Oromia region (13.63% (95% CI: 3.54, 23.73)). The difference in effects by region might be attributed to the context, respondents' educational backgrounds, duration of intervention, and residence.

The pooled DID percentage effects of nutrition intervention on the DDS among pregnant women who had received equal to or greater than 5 months was high (44.70% (95% CI: 23.46, 106.87)) as compared to the duration of intervention less than 5 months (29.61% (95% CI: 8.35, 50.88)). Similarly, pregnant women who had received an intervention for 5 months and above have a higher pooled mean DID effect (0.98 (95% CI: 0.41, 2.37)) than the corresponding (0.68 (95% CI: 0.49, 0.89)). This finding could be related to the fact that longer, more concentrated, committed, and continuous interventions help the respondents to adapt and practice the intervention daily.

In line with the current study, the effect of nutrition intervention on DDS among the respondents were 14% in Tanzania (Blakstad et al. 2021), 12.5% in rural Malawi (Ziyenda Katenga‐Kaunda et al. 2020), 20% in Malawi (Katenga‐Kaunda et al. 2021), 30% in Bangladesh (Nguyen et al. 2017), and 22% in Bangladesh (Nguyen et al. 2018). The possible justification for this result may be due to the extensive nature of the nutritional education intervention, which includes the prospective decision‐makers of the family (the husband) and the peer, which could account for the improvement in dietary DDS among pregnant women. Similarly, nutrition education and counseling can help women understand the need for a diverse diet throughout pregnancy and promote ongoing efforts to maintain a broad diet. Furthermore, health promotion‐based educational interventions can be effective in raising awareness, improving understanding of hazards, removing barriers to good behavior, and, ultimately, improving women's health and nutritional performance during pregnancy (Chowdhury et al. 2017; Nair et al. 2016; Nguyen et al. 2018; Girard and Olude 2012).

In contrast to the current study, the effect of nutrition intervention on DDS among pregnant women was −1.5% in India (Nguyen et al. 2021). This finding difference may be attributed to the straightforward approach of nutritional education used in this intervention, which was conveyed in a culturally acceptable way; engagement of the husbands and selected peers may help them remember and practice it; and incorporating health behavior models and theories into behavior change communication improves food habits. Increased behavioral adjustments toward DDS may suggest that the intervention was effective in inspiring positive ideas and expectations about good nutrition (Tsegaye et al. 2022; Bartholomew and Mullen 2011; Shuremu et al. 2023; Darmon and Drewnowski 2008).

Male and peer involvement during the intervention (Tsegaye et al. 2022; Kuma et al. 2023), home gardening (Kuma et al. 2023), providing nutrition education training packages for health care providers (Omer et al. 2020), having received the intervention, educational status, and a wealth index (Beressa et al. 2024) were all predictors of the effect of nutrition intervention on DDS among pregnant women. This study found that incorporating males and peers in nutrition education interventions helped to improve DDS. The findings also showed that couples should be targeted when making nutrition education interventions. Updated and continued provision of comprehensive in‐service nutritional training for healthcare providers working in antenatal care (ANC) units improved the dietary diversity status of the respondents (Omer et al. 2020). Training women in domestic gardening and nutrition enhances their dietary diversity and consumption of nutrient‐dense foods. Educated women may have better career possibilities and wealth, which could enhance their consumption of a variety of meals. Mothers from the wealthiest households may have a well‐diversified diet (Tsegaye et al. 2022; Haque et al. 2024; Paramashanti et al. 2022; Pradhan et al. 2018).

## 5 Conclusion and Recommendation

Nutritional interventions for pregnant women had a positive effect on DDS. Interventions based on health belief models (HBM) and the theories of planned behavior (TPB) have significantly improved the DDS of pregnant women. Male and peer involvement during the intervention had a significantly greater positive influence on pregnant women's dietary diversity than controls. Male and peer involvement throughout the intervention, home gardening, having received the intervention, educational status, and a wealth index were all predictors of the effect of nutrition intervention on DDS. Home gardening has been identified as a promising technique for improving DDS. A comprehensive in‐service nutrition education and counseling package improve how ANC health care providers interact with pregnant women and convey nutrition messages during ANC consultation.

Therefore, efforts should be made to promote nutrition intervention utilizing the HBM and TPB, targeting couples in constructing nutrition education interventions. The recommendation suggests that home gardening programs could assist in creating wide‐ranging nutrition and health gains in rural farming areas. Health care providers working in ANC units have complete in‐service nutritional training, such as supportive monitoring and supervision, and well‐prepared nutritional guidelines for pregnant women for the provision of adequate nutrition education to the pregnant women.

## 6 Limitation of the Study

Great heterogeneity, the presence of publication bias, and the small number of studies retrieved in Ethiopia were the limitations of the study. Conducting two pooled results is one of the strengths, and other strengths are similar to the strengths of the included studies.

## Author Contributions

M.A.B. performed the following activities: writing the initial draft of the manuscript, designing the procedure, selecting studies, data extraction, analysis, and interpretation of the data. Data extraction, quality assessment, statistical analysis, and manuscript draft revision were all completed by M.B.S., S.D.C., W.M.T., Y.M., Y.W., Z.A.G., Z.A., A.T.A., A.G., G.G.K., and K.W.M. M.A.B. and Y.M. prepared the final draft of this work. After reading the work, all authors provided their final approval.

## Ethics Statement

The protocol for the systematic review and meta‐analysis was registered under the registration ID CRD42024539357 with PROSPERO, and all procedures followed the Declaration of Helsinki.

## Consent

The authors have nothing to report.

## Conflicts of Interest

The authors declare no conflicts of interest.

## Data Availability

All relevant data are available in the manuscript without any restrictions.
